# Coloured Saliva

**Published:** 1844-06-01

**Authors:** 


COLOURED SALIVA.

The saliva sometimes presents a blueish tinge, under the continued use of acetate of lead; it has been known to assume a deep blue tint when indigo has been largely administered, and has yielded indigo on the application of heat to the dried residuum. The heat required is about 550°, and the indigo sublimes in acicular crystals. In the advanced stages of fever and purpura, Dr. Wright has also seen it of a deep blue colour, resulting as he believes, (for he did not examine it chemically) from the presence of Prussian blue, due to the spontaneous decomposition of the blood. The saliva often exhibits a great variety of colours, from a light red, or brown, to a deep purple, or black, in cachectic diseases, and in putrid fevers. These shades of colour, Dr. Wright also refers to the presence of blood in various stages of decomposition, though both the microscope and chemical analysis usually fail in recognising any of the distinctive characteristics of genuine blood. In the mesenteric diseases of children, in various forms of scrofula, and in malignant diseases affecting the abdominal viscera in elderly people, the saliva is not unfrequently of a fawn colour, and excessively offensive to the smell. In these cases, the colouring matter is generally due, either to the ptyaline being depraved, or to the presence of an oily fluid. A casual discolouration of the saliva often arises from the use of certain medicines, such as rhubarb, cochineal, &c., which may sometimes continue long after the use of the medicine has been discontinued.—*Ibid.*

